# Lower Extremity Fillet Flap With Plantar Skin for Reconstruction of Pressure-Related Ulceration in a Patient With Transverse Myelitis

**DOI:** 10.7759/cureus.114554

**Published:** 2026-08-14

**Authors:** Alexander Ponce, McKinley R Seal, Alexandria Gudeman, Thomas M Jones, Derek Culnan

**Affiliations:** 1 General Surgery, Central Surgical Associates, Jackson, USA; 2 General Surgery, William Carey University College of Osteopathic Medicine, Hattiesburg, USA; 3 Plastic Surgery, University of Mississippi Medical Center, Jackson, USA; 4 Surgery, Central Surgical Associates, Jackson, USA

**Keywords:** osteomyelitis, plantar skin, reconstructive surgery, skin graft, wound care

## Abstract

Transverse myelitis is a rare, acquired inflammatory neurologic disorder that can result in chronic disability and secondary complications, including decubitus ulcers. These ulcers may serve as portals for infection and can progress to osteomyelitis, particularly when involving high-pressure areas. We present the case of a 45-year-old female with chronic transverse myelitis who developed a stage 4 perineal, ischial, and femoral decubitus ulcer complicated by acute-on-chronic osteomyelitis of the pelvis and femur. The patient underwent a left lower extremity amputation with preservation of the soft tissues as a myocutaneous turn-up fillet flap, which was utilized to reconstruct and cover the extensive ulcer defect covering the chronically exposed femur and pubic rami. Due to its durability and resistance to pressure and shear forces, the plantar skin of the amputated foot was used to reconstruct the ischial region, an area subject to significant mechanical stress. This case highlights a severe complication of transverse myelitis and introduces a novel reconstructive approach using a fillet flap with plantar skin for the coverage of high-pressure decubitus ulcers. Further studies are needed to evaluate long-term outcomes in the potential broader application of this reconstructive technique.

## Introduction

Transverse myelitis is an acquired inflammatory neurologic disorder that targets the spinal cord [1]. An external trigger, such as an autoimmune disorder, a rheumatologic disorder, an infection, or intravenous drug use, activates the immune system and leads to inflammation within the central nervous system [2,3]. Perivascular monocytic and lymphocytic infiltrations, demyelination, and axonal injury will be seen at the site of the lesion [1,3]. Transverse myelitis can present with acute back pain with radiculopathy, bilateral extremity weakness, bladder and bowel incontinence, and bilateral sensory deficits [4]. On average, the symptoms of transverse myelitis begin to subside between three and six months. Patients may recover with no lasting deficits, have a minimal to moderate degree of permanent disability, or remain permanently disabled, as in this case of a paraplegic patient [1].

Potential complications of transverse myelitis are dependent on the severity and duration of the disease. Common symptoms for both acute and chronic transverse myelitis involve the function of the bladder and bowels, such as bladder dysfunction, constipation, gastroparesis, or fecal incontinence [5]. Another common complication of chronic transverse myelitis is the development of decubitus ulcers [1]. A decubitus ulcer is a wound that starts superficially in the skin due to external pressure that has been applied over an excessive amount of time [6]. The ulcer may continue to enlarge and deepen into other tissue layers without treatment and counteractive measures [6]. Local bacterial or systemic infections can occur with decubitus ulcers [6]. Preventative measures involve promotion of movement, pressure avoidance, and pressure reduction/distribution [6]. Dressing and debriding the ulcers, and antibiotic treatment are essential for all classifications of ulcers. A flap may be the optimal treatment if the decubitus ulcer is severe, there is no potential for healing, and the patient is a good surgical candidate [6].

Here, we present the case of a 45-year-old woman who was paraplegic as a sequel of transverse myelitis as a teen. The patient developed a decubitus ulcer and osteomyelitis as a complication of her transverse myelitis. This case highlights a rare occurrence of chronic transverse myelitis and a severe complication of the disease. It introduces a strategic flap procedure that utilized the robust plantar skin of the foot as well as the lower extremity musculature to cover the large ulcer area and resist the pressure and shear forces generated by using her wheelchair and transferring in and out of it. After a successful surgery, the patient underwent several additional operative debridements and procedural revisions. Postoperatively, the skin flap has remained intact with no complications.

## Case presentation

**Figure 1:**
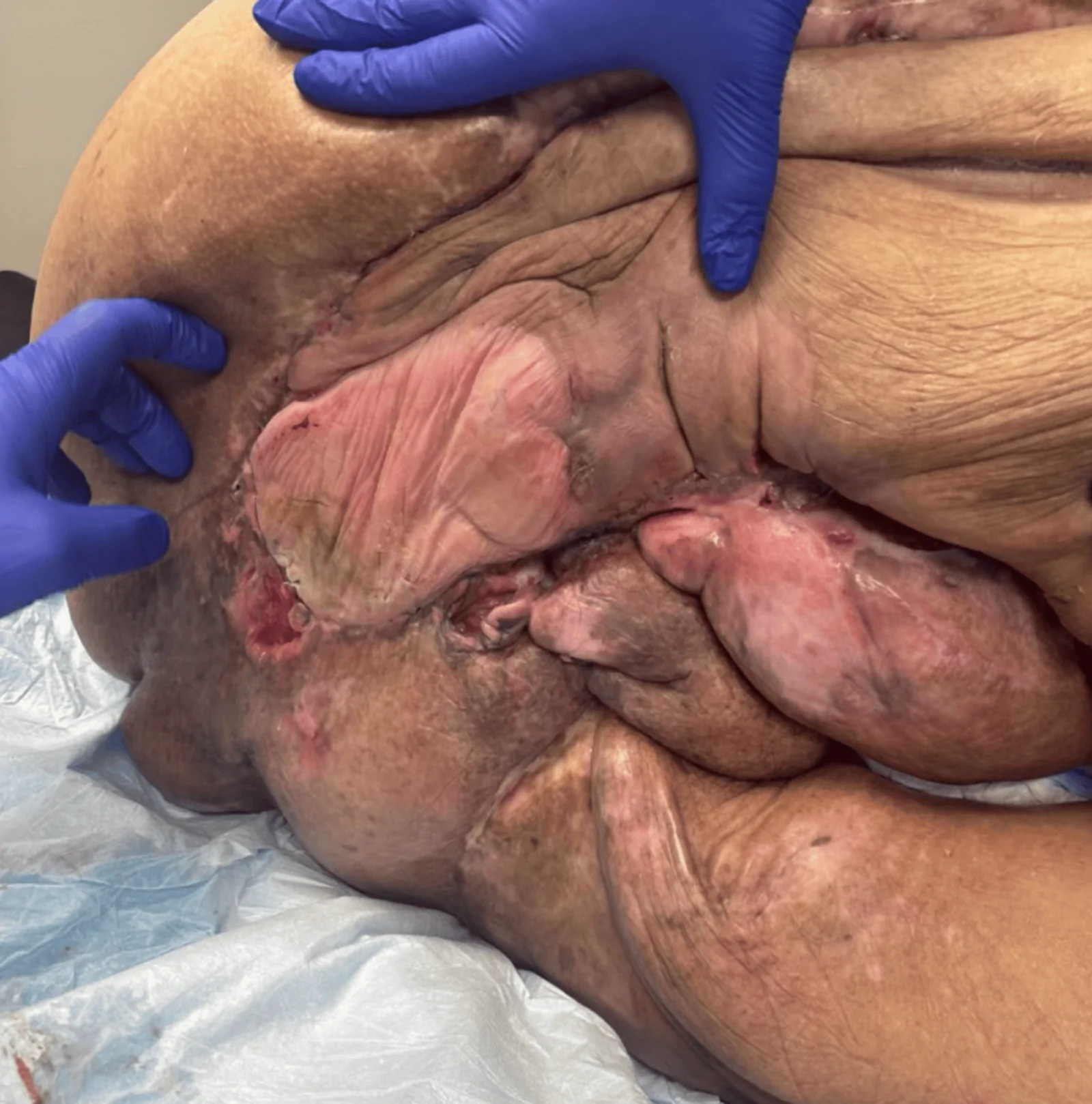
Some ulceration at the right side of the anus and at the medial aspect of the right thigh. See this detailed in Figure 4.

**Figure 2:**
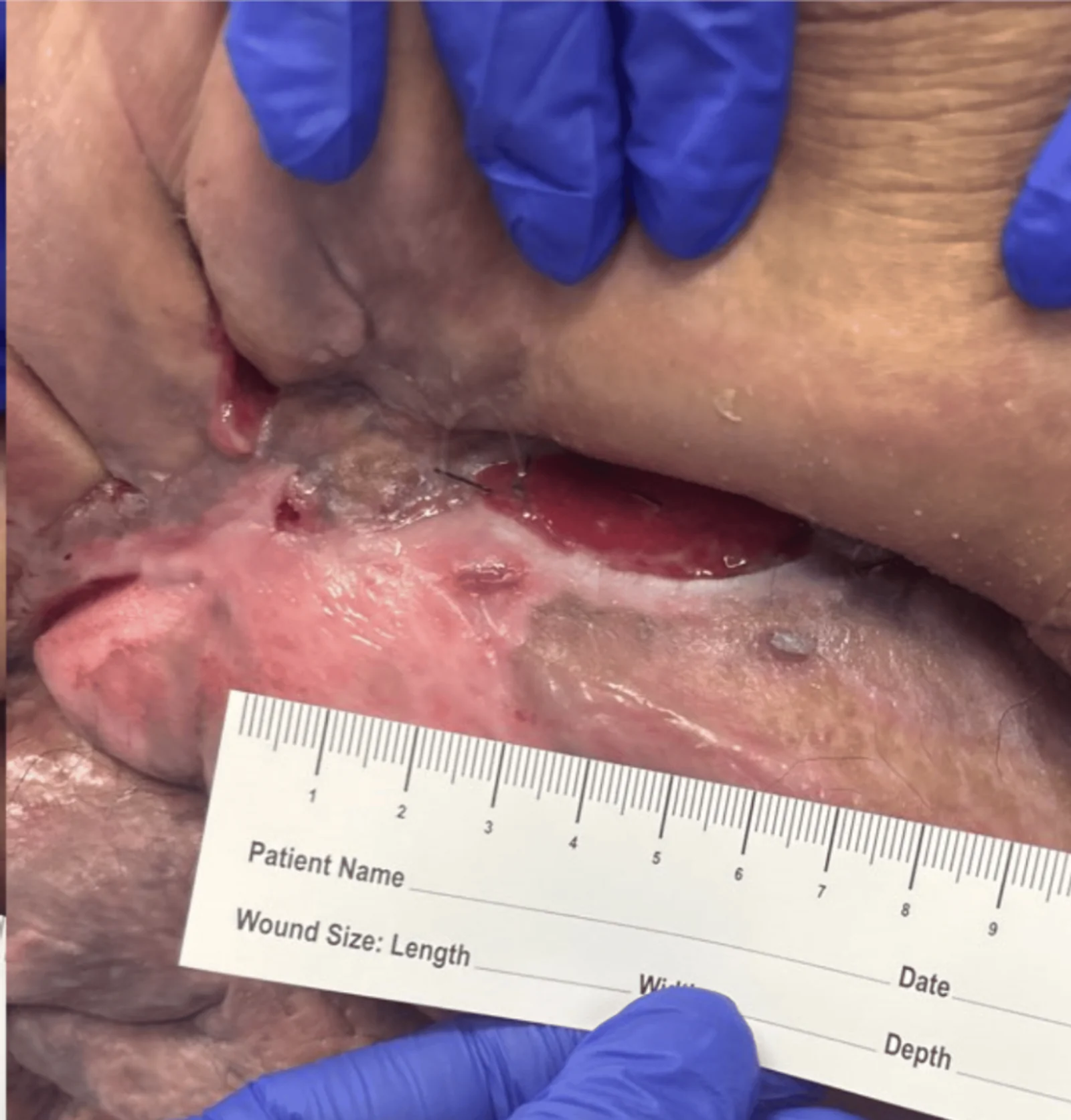
Residual ulcer at the edge of the flap on the proximal medial thigh.

In January 2026, a 45-year-old female with a past medical history of transverse myelitis resulting in paraplegia presented with a longstanding right-sided stage IV perineal, ischial, and femoral decubitus ulcer complicated by chronic osteomyelitis. Given the chronic nature and severity of the ulcer, as well as the complexity of the procedure, the flap edges were loosely approximated to facilitate drainage, minimize the risk of fluid collection and infection, and allow demarcation of any nonviable tissue. A staged reconstruction was planned with the expectation of returning to the operating room for serial debridement and definitive flap revision over the following several weeks (Figures 1, 2). Postoperatively, the patient was maintained on a sand bed, intravenous antibiotics, and negative-pressure wound therapy using a vacuum-assisted closure device. She was subsequently transferred to a long-term acute care facility to continue physical therapy and complete her course of intravenous antibiotics. During this time, she underwent several additional operative debridements and minor revision procedures. She was later discharged to an inpatient rehabilitation facility, where she worked on transfer techniques while minimizing shear forces. At her two-week postoperative follow-up after returning home, the flap was found to be well incorporated, with only a few small areas of residual wound separation along the edges.

## Discussion

Chronic transverse myelitis resulting in pressure ulcer formation is not common, as most patients with transverse myelitis do not have paraplegia of the lower extremities. An estimated 50% have complete paralysis, resulting in a significant decrease in function and daily life [7]. Patients with residual paralysis and sensory loss are at an increased risk of secondary complications most commonly associated with immobility [8]. The loss of protective sensation and the impaired ability to reposition due to motor, sensory, and autonomic dysfunction can contribute to prolonged pressure exposure, particularly within the lower extremities and pelvis.

Decubitus ulcers occur via prolonged or severe overloading [9]. This overloading leads to deformation, inflammation, and eventual necrosis [9]. Shearing forces during transfer also contribute to ulcer formation. In neurologically impaired patients, these wounds can become chronic due to continued pressure and impaired wound healing [10]. Chronic ulceration provides a route for bacterial invasion and contiguous spread to bone, leading to the development of osteomyelitis [11].

Once osteomyelitis has been diagnosed, management becomes more complex [12]. Traditional management of patients with decubitus ulcers consists of local wound care, serial debridement, antibiotics, and frequent position changes to offload pressure [10]. Although management of osteomyelitis associated with pressure ulcers varies, treatment generally consists of intravenous antibiotics and debridement of the affected bone, guided by bone cultures whenever possible to confirm the diagnosis [11]. Beyond the management of osteomyelitis, surgical reconstruction is often required in patients with advanced disease [13]. The most common surgical reconstruction interventions are skin grafting and flap coverage [13]. However, these approaches may be limited in efficacy when involving high-pressure or weight-bearing regions of the body. Graft durability and long-term wound stability are challenged by ongoing mechanical stress, particularly common in patients with persistent neurologic deficits [14]. Nutrition, infection, and other comorbidities also contribute to the perpetuation of these ulcers.

This patient’s case was notable for the prolonged nature of this large decubitus ulcer, recurrence of infection, and significant soft-tissue and bony loss that continued to worsen after a large traditional rotational flap eventually broke down again, exposing her pelvic and proximal femur and then extending down the femur to the popliteal fossa.

Her fevers and bouts of nausea began to occur with increasing frequency despite debridement, aggressive wound care, and multiple rounds of antibiotics. After prolonged discussions of risks, benefits, and alternatives with the patient, plastic surgeon, and infectious disease physician, it was deemed necessary to sacrifice the right lower leg as a myocutaneous flap to salvage the proximal thigh and pelvis to preserve her mobility, stability, and freedom from recurring and ongoing infections. Conventional reconstructive options were limited by previous failed flaps and considered suboptimal given the patient’s underlying paralysis and continued risk of pressure-related injuries. Therefore, alternative tissue sources capable of withstanding increased mechanical demands were considered.

Plantar skin possesses unique structural characteristics that make it well suited for reconstruction in high-pressure environments when adequately perfused [15]. Compared with other sections of skin on the human body, plantar skin is thicker, more highly keratinized, and therefore inherently adapted to resist shear forces and repetitive loading [16]. In the setting of an amputation or other means of availability, this tissue may be readily available and can be repurposed to provide durable coverage in areas at risk for recurrent breakdown [17]. In this case, plantar skin was used for reconstruction of the high-wear area, and the highly vascular muscular portion of the leg was used to cover the exposed residual femur.

Management of pressure-related ulcers in patients with chronic neurologic impairment remains challenging due to persistent immobility, sensory loss, and continuous mechanical stress. While standard reconstructive options such as skin grafts and flap coverage are commonly employed, their durability may be limited in high-pressure or weight-bearing regions, particularly in patients with irreversible deficits [14]. This case demonstrates how consideration of tissue-specific biomechanical properties may inform reconstructive decision-making in complex scenarios, especially when conventional options are unlikely to provide long-term wound stability.

## Conclusions

This case highlights the potential role of a plantar skin flap as part of a fillet flap as a reconstructive option in select patients with neurologic impairment and pressure-related wounds. In this patient, plantar skin provided durable coverage of the pressure-prone wound following amputation. Limb sacrifice for myocutaneous flap coverage is complex and should be reserved for select patients when simpler options are not available or have failed, yet the patient is healthy enough to undergo an extensive surgery. The cost to the patient of losing a limb must be weighed against what function or benefit can be salvaged by closing the ulcer or covering the exposed bone. While further evaluation is needed to assess long-term outcomes and broader applicability, plantar skin grafting may represent a valuable adjunct to existing reconstructive strategies, particularly in post-amputation settings where durable, pressure-resistant coverage is essential.
